# Exploring the transfer of recent plant photosynthates to soil microbes: mycorrhizal pathway vs direct root exudation

**DOI:** 10.1111/nph.13138

**Published:** 2014-11-10

**Authors:** Christina Kaiser, Matt R Kilburn, Peta L Clode, Lucia Fuchslueger, Marianne Koranda, John B Cliff, Zakaria M Solaiman, Daniel V Murphy

**Affiliations:** 1 Soil Biology and Molecular Ecology Group, School of Earth and Environment, Institute of Agriculture, The University of Western AustraliaCrawley, WA, 6009, Australia; 2 Department of Microbiology and Ecosystem Science, Division of Terrestrial Ecosystem Research, Faculty of Life Sciences, University of ViennaAlthanstrasse 14, Vienna, A-1090, Austria; 3 Centre for Microscopy, Characterisation and Analysis, The University of Western AustraliaCrawley, WA, 6009, Australia

**Keywords:** arbuscular mycorrhizal (AM) fungi, belowground carbon allocation, hyphosphere, mycorrhizosphere, NanoSIMS, priming effect, recent photosynthates, root exudates

## Abstract

- Plants rapidly release photoassimilated carbon (C) to the soil via direct root exudation and associated mycorrhizal fungi, with both pathways promoting plant nutrient availability. This study aimed to explore these pathways from the root's vascular bundle to soil microbial communities.
- Using nanoscale secondary ion mass spectrometry (NanoSIMS) imaging and ^13^C-phospho- and neutral lipid fatty acids, we traced *in-situ* flows of recently photoassimilated C of ^13^CO_2_-exposed wheat (*Triticum aestivum*) through arbuscular mycorrhiza (AM) into root- and hyphae-associated soil microbial communities.
- Intraradical hyphae of AM fungi were significantly ^13^C-enriched compared to other root-cortex areas after 8 h of labelling. Immature fine root areas close to the root tip, where AM features were absent, showed signs of passive C loss and co-location of photoassimilates with nitrogen taken up from the soil solution. A significant and exclusively fresh proportion of ^13^C-photosynthates was delivered through the AM pathway and was utilised by different microbial groups compared to C directly released by roots.
- Our results indicate that a major release of recent photosynthates into soil leave plant roots via AM intraradical hyphae already upstream of passive root exudations. AM fungi may act as a rapid hub for translocating fresh plant C to soil microbes.

## Introduction

A significant proportion of plant photosynthates is transported belowground shortly after photoassimilation and subsequently released to soil microbes (Dilkes *et al*., 2004; Bahn *et al*., 2009; Mencuccini & Hölttä, 2010). This release can be through direct exudation from the surface of fine roots or by transfer to the extraradical mycelium of mycorrhizal fungi (Jones *et al*., 2004, 2009; Drigo *et al*., 2010). Both root exudation and transfer to mycorrhizal fungi occur rapidly after photosynthesis, ranging from a few hours in grasses to a few days in trees (Johnson *et al*., 2002; Dilkes *et al*., 2004; Kuzyakov & Gavrichkova, 2010). Root exudation stimulates microbial decomposition of soil organic matter, which in turn improves nutrient availability along the rhizosphere (Kuzyakov, 2010; Bird *et al*., 2011; Philippot *et al*., 2013). Carbon (C) transfer to mycorrhizal fungi benefits the plant through direct nutrient transfer from the fungal hyphal network (Bever *et al*., 2009; Fellbaum *et al*., 2011; Kiers *et al*., 2011). In both cases, the plant's investment in belowground C allocation is rewarded with increased nutrient availability, in particular nitrogen (N) and phosphorus (P) (Hodge & Storer, 2014).

Mycorrhizal fungi improve a plant's access to nutrients and water by extending its range into soil areas that are not accessible by roots and to nutrient-rich soil hot-spots via a vast network of extraradical mycelium. Current hypotheses propose that mycorrhizal hyphae also stimulate surrounding soil microbes by exuding labile C, and thus increase local nutrient availability in the hyphosphere (Hodge *et al*., 2010; Cheng *et al*., 2012; Jansa *et al*., 2013). Direct evidence for the transfer of plant-derived C from mycorrhizal hyphae to surrounding soil microbes is however still lacking (Jansa *et al*., 2013), although some studies have shown that arbuscular mycorrhizal (AM) hyphae potentially exude plant-derived C (Johansson *et al*., 2004; Toljander *et al*., 2007; Cheng *et al*., 2012). Importantly, mycorrhizal fungi may be able to spatially shift the prolific C transfer to soil microbes via root-inaccessible places, which would be highly beneficial for plant nutrient supply (Cheng *et al*., 2012; Herman *et al*., 2012; Nottingham *et al*., 2013).

Although there are likely to be different returns for the plant on investment of C released by direct root exudation vs C traded to mycorrhizal fungi, not much is known about the pathways and crossroads of these two C flows in the plant root (Badri & Vivanco, 2009). It is thought that the largest proportion of root-exudate C is lost passively, driven by the large C concentration gradient between the root cytoplasm and the apoplast (Farrar *et al*., 2003; Jones *et al*., 2009). However, in most of the root area the endodermal layer (i.e. Casparian strip) would block diffusion of phloem-released C to the root cortex and further to the soil solution. Exudates are thus thought to ‘leak’ primarily from the relatively small immature root zones close to the root tip. Here sugars can diffuse from the protophloem into the apoplast and from there further to the soil solution, without being blocked by endodermal cells (Farrar *et al*., 2003). AM fungi, on the other hand, colonise roots predominately behind the root hair zone in mature roots that exhibit a developed phloem and endodermal layer (Smith & Smith, 2011). Plant C is thought to be transferred to the fungus as sucrose or hexose across mycorrhizal intraradical structures (hyphae or arbuscules in the root cortex), and transported as glycogen or triacylglycerol through the large extraradical hyphae network (Bago *et al*., 2002, 2003; Parniske, 2008). This symbiotic interface comprises plasma membranes from both fungus and plant, separated by an apoplastic compartment (Parniske, 2008; Smith & Smith, 2011). In exchange, fungal hyphae are thought to take up ammonium (NH_4_^+^), nitrate, amino acids and phosphate from the soil solution. These are most likely transported as arginine or polyphosphate granules, before being transferred to the plant as NH_4_^+^ or phosphate (Govindarajulu *et al*., 2005; Jin *et al*., 2005; Parniske, 2008). Improving knowledge on C and N flow pathways through roots and mycorrhizal fungi is a key step in understanding possible regulations and consequences of plant belowground C allocation. However, this has been hampered by the lack of suitable methods for the *in situ* tracing and quantification of C and N flow at the subcellular (i.e. micron) scale, especially at the root–mycorrhiza interface.

Stable isotope labelling experiments using ^13^C and ^15^N enable C and N to be traced through plant and soil pools (Olsson & Johnson, 2005; Paterson *et al*., 2009). However, traditional mass spectrometry analysis is based on bulk plant or soil samples and cannot provide information on cellular and subcellular C and N dynamics. Yet it is precisely at the subcellular and root–soil-microbial interface that studies need to be conducted if we are to elucidate the transfer pathways involved. NanoSIMS does enable simultaneous imaging and isotopic discrimination of stable isotopes (e.g. ^13^C vs ^12^C and ^15^N vs ^14^N) at a subcellular spatial scale (*c*. 100 nm resolution) (Kilburn *et al*., 2010). NanoSIMS techniques have been applied to investigate ^15^N flow through microbes, protozoa, and animal cells (Foster *et al*., 2011; Pernice *et al*., 2012; Woebken *et al*., 2012), as well as rhizosphere bacteria and plant tissue (Clode *et al*., 2009; Jones *et al*., 2013). Application of this technique to explore ^13^C flows from photosynthesis through plant tissues is only just emerging, as these C molecules have proved difficult to preserve during the preparation required for NanoSIMS (Clode *et al*., 2007; Bougoure *et al*., 2014).

Here, our specific aim was to elucidate the pathways through which recently photoassimilated C is transferred from plant roots to soil microbes and the possible role of AM fungi in this process. Our research questions were: by what routes is recently photoassimilated C translocated from the vascular bundle to the soil in an AM-colonised wheat root? Are these routes linked to those for NH_4_^+^ taken up from the soil solution? What role do AM fungi have in the transfer of recently assimilated plant C to the soil microbial community? And, does N addition to roots or mycorrhizal hyphae affect the amount of recently assimilated plant C released to the soil? To address these questions we (1) used NanoSIMS to visualise the flow of ^13^C and ^15^N through wheat roots and their associated intraradical mycelium. For this, plants were exposed to both a ^13^CO_2_ enriched atmosphere and a pulse of ^15^NH_4_^+^ applied to the soil. (2) We traced the fate of recently plant-assimilated ^13^C in both root- and hyphae-associated microbial communities via phospholipid fatty acid (PLFA) and neutral lipid fatty acid (NLFA) stable isotope probing and in dissolved organic carbon (DOC) pools via isotope-ratio mass spectrometry.

## Materials and Methods

### Experimental set-up: plant–root–mycorrhiza system in split boxes

Wheat plants (*Triticum aestivum* L. var. ‘Wyalkatchem’) inoculated with AM fungi were grown in split-boxes (Supporting Information Fig. S1). Each split-box consisted of two compartments (dimension: 8.5 × 7 × 10 cm) that were separated by an assemblage of two membranes (mesh size: 30 μm) encapsulating a solid 1.5-mm-thick, wide-meshed plastic grid in between. The grid established a small void between compartments to prevent solution flow (Zhang *et al*., 2010). Mycorrhizal hyphae, but not wheat roots, were able to grow through these membranes. Each compartment was filled with 500 g of fresh soil. The soil was a coarse-textured agricultural soil routinely used for wheat production in rotation with legumes (88% quartz sand) from Mingenew, Western Australia (29°19′N, 115°44′E; samples collected from the 0 to 10 cm Ap horizon: 10.3 g organic C kg^−1^ and 0.9 g total N kg^−1^; sieved < 4 mm). Before use, the soil had been inoculated with 25 g of clipped fine root biomass from bioassay pots growing AM-rich subterranean clover (*Trifolium subterraneum*). This fostered the establishment of plant AM colonisation. Germinating wheat seeds were planted in one compartment of each box (‘root compartment’ i.e. contains both plant roots and mycorrhizal hyphae). The other compartment only hosted mycorrhizal hyphae and was termed ‘hyphae compartment’. Unplanted control split-boxes did not receive seeds in either compartment.

### Double ^13^CO_2_ and ^15^NH_4_^+^ pulse labelling experiment

In order to simultaneously trace the flow of recent photosynthates and soil inorganic N through the plant root mycorrhizal system, we conducted a double ^13^CO_2_ and ^15^NH_4_ labelling experiment. Split-boxes with 4-wk-old wheat plants and unplanted controls were subjected to an enriched ^13^CO_2_ atmosphere for 8 h. ^13^CO_2_-labelling was carried out in three 155-l gas-tight perspex chambers, each containing up to 35 randomly allocated split boxes. Chambers were situated in a temperature-regulated glasshouse at 20°C under natural daylight conditions. ^13^CO_2_ labelling began in the morning of a cloudless, sunny day in April. After the initial headspace CO_2_ concentration in each chamber had decreased to 140 ppm, we added 40 ml ^13^CO_2_ (99 atom% ^13^C; Sigma-Aldrich), equivalent to 340 ppm, into each chamber with a gas-tight syringe. This was followed by regular injections of 30 ml ^13^CO_2_, equivalent to 250 ppm, every 30 min. This approach repeatedly restocked photosynthetically driven CO_2_ depletion in each chamber (average CO_2_ depletion had been determined in a pre-experiment and was in the range of 8 ppm min^−1^). This resulted in a quick rise of ^13^C abundance in the CO_2_ in the chambers (95 atom% by the 3rd injection) with CO_2_ concentrations fluctuating between 250 and 500 ppm over 30-min intervals. As split-boxes of all harvests and treatments were distributed randomly across the three chambers, all three chambers needed to be opened after 4 h to take out the split-boxes for the first harvest. This was done quickly to keep air exchange to a minimum. However, chamber ^13^C concentrations dropped to natural abundance after the first harvest and could only be re-established with a subsequent ^13^CO_2_ injection 30 min later. The atom% ^13^C of CO_2_ in the chambers was measured with a Delta XL isotope ratio mass spectrometer connected to a GasBench II (Thermo Fisher, Bremen, Germany). Three planted split-boxes were also kept in a separate room (to avoid ^13^CO_2_ contamination). These served as unlabelled (natural abundance) controls for calculating ^13^C excess in plant biomass, microbial biomass, DOC, individual NLFA and PLFAs, and the NanoSIMS-measured compartments within fine roots.

Two hours after the start of ^13^CO_2_ labelling, 15 ml of 1 mM (^15^NH_4_)_2_SO_4_ (99 atom% ^15^N; Sigma-Aldrich) was added drop wise into either the root- or the hyphae-compartment of the split-boxes. This was achieved with a long needle through pre-installed rubber septa in the upper cover of the perspex chamber (one septum aligned above each split box).

In order to evaluate the flow of recently photoassimilated plant C through both root and hyphae-associated soil, we measured ^13^C of DOC, microbial biomass, and NLFAs and PLFAs of soil samples. These were from both root- and hyphae-compartments harvested 4, 8, and 24 h after the start of the 8 h ^13^CO_2_ labelling period. This corresponded to 2, 6 and 22 h (respectively) after ^15^N addition (*n *=* *5 replicate split-boxes for each time point and treatment). Unplanted control split-boxes served as a control to assess the possibility of direct (i.e. photo- or chemo-autotrophic) CO_2_ fixation by soil microbes. These were harvested 8 h after the start of ^13^CO_2_ labelling. In addition, samples of fine roots for NanoSIMS analysis were taken from labelled and control (*n *=* *3) split-boxes 8 h after the start of the ^13^CO_2_ labelling period. AM fungal colonisation of roots was assessed by light microscopy after staining with Trypan blue using a gridline intercept method (Newman, 1966).

### Phospholipid and neutral fatty acids

PFLAs and NLFAs were extracted from soil with a mixture of chloroform, methanol and citrate buffer (1 : 2 : 0.8 v/v/v). PLFAs were separated from NLFAs using silica bonded columns. After fractionation, both PLFAs and NLFAs were converted to methyl esters by alkaline methanolysis (Frostegård *et al*., 1991). Dried fatty acid methyl esters (FAME) were re-dissolved in isooctane, and concentrations and C isotope ratios were determined by a Trace Ultra GC (Thermo Fisher) interfaced with an Isotope Ratio Mass Spectrometer (Delta V Advantage; Thermo Fisher) via a combustion unit (GC combustion II/TC; Thermo Fisher). A FAME mixture (Supelco, nrs 47080-U and 47885-U) was used as a qualitative standard. For calculation of FAME concentrations and isotopic ratios an internal standard (19:0) was used. In addition, isotopic ratios were corrected for ^13^C : ^12^C ratio of C added during the methanolysis. Fatty acids i15:0, a15:0, i16:0, i17:0, a17:0 were used as indicators of Gram-positive bacteria, cy17:0 as an indicator of Gram-negative bacteria, 14:0, 15:0 and 17:0 as indicators for bacteria in general, and 18:1ω9c and 18:2ω6,9 as general markers for fungi (Zelles, 1999; Leckie, 2005; Joergensen & Wichern, 2008; Kaiser *et al*., 2010). Interpretation of ^13^C enrichment of the fungal biomarkers 18:1ω9 and 18:ω6,9 was undertaken with care as these biomarkers are known to also occur in plants. Earlier work has shown that the contribution of plant-borne 18:2ω6,9 from roots that remain in sieved soil compared to the total 18:2ω6,9 is negligible (Kaiser *et al*., 2010). However, as the present approach involves strong labelling of the plant with ^13^C, the expected contribution of plant-borne ^13^C-labelled PLFAs to total ^13^C-labelled PLFAs in sieved soil may be higher. We thus excluded PLFAs 18:1ω6 and 18:2ω6,9 from part of our analysis to get a more reliable pattern of ^13^C in soil microbial communities. Although PLFAs 16:1ω5 and 18:1ω7 are frequently used as a signature for AM fungi, they are also known to occur in Gram-negative bacteria (Ruess & Chamberlain, 2010). As our analysis showed that PLFA 18:1ω7 occurred in similar amounts in unplanted controls, whereas 16:1ω5 was absent (Table1), we only used the latter as a PLFA marker for AM fungi. In contrast to their PLFA counterpart, NLFAs 16:1ω5 and 18:1ω7 are known to occur specifically in AM fungi storage compounds and not in other microbial groups, making them excellent biomarkers for AM fungi (Olsson & Johnson, 2005; Drigo *et al*., 2010; Ruess & Chamberlain, 2010). Total PLFA and NLFA concentrations were calculated as mg C g^−1^ dry soil. The amount of C from recent root exudates incorporated into PLFA/NLFAs was calculated as outlined below.

**Table 1:** Concentrations of phospholipid- and neutral fatty acids (PLFAs and NLFAs) in root- and arbuscular-mycorrhizal hyphae-associated split-box compartments planted with wheat (*Triticum aestivum*) and in unplanted controls

Biomarker for	PLFAs/NLFAs used	Root-accessible soil		Hyphae-accessible soil		Unplanted control	
		Mean (SE)	*n*	Mean (SE)	*n*	Mean (SE)	*n*
	PLFAs						
Gram+	i15:0, a15:0, i16:0, i17:0, a17:0,	3.53 (0.23)	15	3.74 (0.38)	14	2.38 (0.30)	6
Gram−	cy17:0	0.51 (0.04)	15	0.52 (0.05)	14	0.64 (0.10)	6
Bacteria	Gram+, Gram−, 14:0, 15:0, 17:0	4.63 (0.30)	15	4.90 (0.51)	14	3.66 (0.46)	6
Fungi	18:1ω9, 18:2ω6,9	1.80 (0.13)	15	1.80 (0.16)	14	1.62 (0.18)	6
Arbuscular mycorrhizal fungi							
	PLFAs						
	16:1ω5	0.24 (0.04)	15	0.29 (0.06)	14	nd (–)	6
	18:1ω7	0.90 (0.08)	15	1.0 (0.10)	14	1.08 (0.13)	6
	16:1ω5/Bacteria	0.05 (0.01)	15	0.05 (0.01)	14	– (–)	
	18:1ω7/Bacteria	0.19 (0.01)	15	0.21 (0.01)	14	*0.30* (0.03)	6
	NLFAs						
	16:1ω5	2.40 (0.34)	14	1.93 (0.22)	15	nd (–)	6
	18:1ω7	0.66 (0.34)	14	0.62 (0.04)	15	nd (–)	6
Microbial biomass							
	Total PLFAs	12.30 (0.82)	15	12.91 (1.17)	14	12.71 (1.23)	6
	Total PLFAs excluding potentially plant-related (18:1ω9, 18:2ω 6,9)	10.50 (0.73)	15	11.10 (1.02)	14	11.10 (1.06)	6

Values are means of *n* split-boxes harvested at any time during the experiment (root- and hyphae-associated soil: Planted split-boxes harvested at 4, 8 and 24 h after ^13^CO_2_-labelling plus unlabelled (planted) control split-boxes; Unplanted control split-boxes: set up at the same time as planted ones but without plants, harvested 8 h after ^13^CO_2_-labelling). AM Fungi, Arbuscular mycorrhizal fungi. SE, standard error; nd, not detectable. All concentrations are in μg C g^−1^ dry soil except for 16:1ω5/Bacteria and 18:1ω7/Bacteria which represent the ratio of AM fungi PLFA to sum of bacterial PLFAs.

### ^13^C in DOC and microbial biomass

In order to estimate DOC and total microbial biomass in root and hyphal compartments, 20 g soil was extracted with 80 ml of 0.5 M K_2_SO_4_ before and after chloroform fumigation (Amato & Ladd, 1988). Dissolved organic C concentration and ^13^C of DOC were measured in aliquots of the extracts on an HPLC (Dionex Corp., Sunnyvale, CA, USA) connected to a Finnigan Delta V Advantage Mass Spectrometer (Thermo Electron, Karlsruhe, Germany) linked by a Finnigan LC-IsoLink Interface (Thermo Fisher) by direct injection (without column, direct mode) at a flow of 0.5 ml ultrapure water min^−1^ (Millipore, Vienna, Austria). Microbial biomass C was calculated from differences in DOC in fumigated and nonfumigated soil samples. The fraction of ^13^C in the microbial biomass C (^13^C_MB_) was calculated by a two-pool mixing model from isotope ratios and DOC concentrations in fumigated and nonfumigated soils:


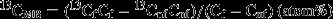
Eqn 1

(^13^C_f_ and ^13^C_nf_, ^13^C-fractions of C in the fumigated and nonfumigated samples (as atom% ^13^C), respectively; C_f_ and C_nf_, absolute concentrations of C (μg C g^−1^ soil) in fumigated and nonfumigated samples, respectively.)

### Calculation of isotopic enrichment in microbial biomass, DOC and individual PLFA/NLFAs

We calculated the excess of ^13^C relative to the unlabelled control in the microbial biomass, the DOC pool, or each individual PLFA/NLFA (APE ^13^C_X_) as:


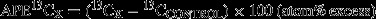
Eqn 2

(^13^C_X,_ measured fraction of ^13^C in the microbial biomass, DOC or individual PLFA/NLFAs; ^13^C_CONTROL_, fraction of ^13^C of the respective unlabelled control (in atom%).)

We calculated the total amount of excess ^13^C (μg C g^−1^ soil) that has been incorporated in the microbial biomass, DOC or each PLFA/NLFA (^13^C_INC_), respectively, as:


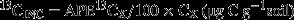
Eqn 3

(C_X_ refers to the C concentration in microbial biomass, DOC or PLFAs/NLFAs, respectively (in μg C g^−1^ soil).)

### Plant C assimilation (EA-IRMS)

Carbon and N concentrations, and isotope ratios of ground plant root and shoot samples were analysed with a PDZ Europa ANCA-GSL elemental analyser interfaced to a PDZ Europa 20-20 IRMS (Sercon Ltd, Cheshire, UK, analysed at UC Davis Stable Isotope facility, Davis, CA, USA).

### Sample preparation for NanoSIMS analysis

**Figure 1:**
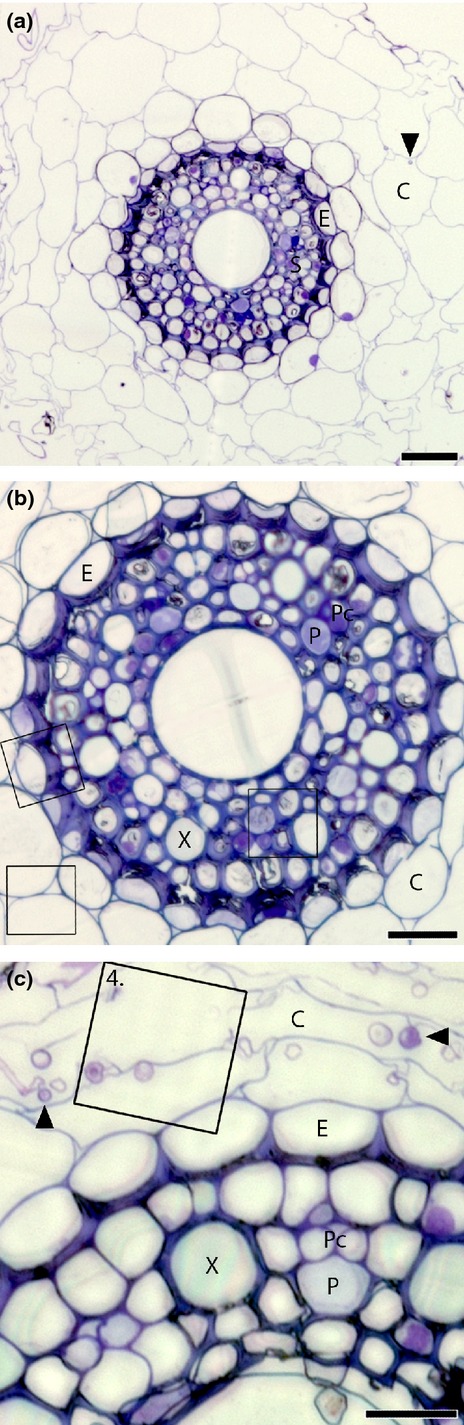
Light micrographs of transverse sections through resin-embedded mature fine roots of wheat (*Triticum aestivum*) associated with arbuscular mycorrhizal fungi, cut dry at 1-μm thickness and stained with toluidine blue, showing cellular structure and examples of typical regions analysed by NanoSIMS. (a) Whole root, highlighting the cortex (C); endodermis (E) and stele (S) cell layers. Mycorrhizal fungal hyphae (black arrows) are also evident in the cortex. (b) Cellular detail within the stele, including xylem (X); phloem (P); and phloem companion cells (Pc); square outlines indicate typical fields of view analysed by NanoSIMS in the cortex (C), endodermal region (E) and stele. (c) Intraradical mycorrhizal hyphae (black arrows) within the root cortex (C). Cell structure within the stele, including xylem (X); phloem (P); and phloem companion cells (Pc) is also evident. The square region outlined is an example of a typical region analysed by NanoSIMS, and in this case is the same region shown in Fig.4. Bars: (a) 50 μm; (b) 30 μm; (c) 20 μm.

Fine roots (diameter < 0.5 mm) from split-boxes (*n *=* *3) that were harvested 8 h after the start of ^13^CO_2_-labelling (i.e. 6 h after ^15^N addition) were dissected and immediately rapid-frozen in N_2_ slush. Samples were freeze-substituted in 10% acrolein in diethyl ether and embedded under vacuum in araldite, based upon the method of Marshall (1980) and described in detail by Kilburn & Clode (2014). All solutions were anhydrous and maintained over a molecular sieve, and embedded samples were stored in a desiccator. Transverse sections from resin-embedded material were cut dry at 1-μm thickness, mounted on glass slides and stained with toluidine blue to identify suitable fields of view for subsequent NanoSIMS analyses (Fig.1). Similar dry-cut sections were prepared for NanoSIMS analysis, but these were mounted directly onto Si wafers, before being coated with 5 nm Au (Notes S1).

### NanoSIMS

*In situ* isotopic mapping was performed using a NanoSIMS 50 (Cameca, Gennevilliers, France), with a 16 keV Cs^+^ primary ion beam. Analyses were performed in multi-collection mode with the trolleys positioned to simultaneously detect the negative secondary ions ^12^C^−^, ^13^C^−^, ^12^C^14^N^−^, ^12^C^15^N^−^ and ^31^P^−^. The mass spectrometer was tuned to high mass resolution of *c*. 9000 (CAMECA definition) to separate the ^12^C^15^N^−^ from the ^13^C^14^N^−^ peak on mass 27 and the ^13^C^−^ from the ^13^C^1^H^−^ peak on mass 13 using an entrance slit of 30 μm, an aperture slit of 200 μm, and a 10% reduction in the signal at the energy slit.

For secondary ion imaging, the primary current was set to *c*. 20 pA to optimise the secondary ion signal using a 750-μm primary aperture (D1), giving a spot size of *c*. 200 nm. Images were acquired by rastering the beam over an area 30 × 30 μm square, at a resolution of 256 × 256 pixels, giving a pixel size of 117 nm. Optical micrographs of sections were used to navigate around the sample. Image data consist of the total number of counts for a given secondary ion species recorded on each pixel, with count times kept constant at 60 ms per pixel. All areas were implanted to the same ion dose by the primary beam before each acquisition to remove surface contamination and to enhance the generation of secondary ions.

Images were processed using the OpenMIMS data analysis software (National Resource for Imaging Mass Spectrometry http://nrims.harvard.edu) for the freeware package ImageJ (National Institutes of Health, Bethesda, MD, USA). Images were corrected for detector dead time (44 ns) on individual pixels before any other data processing. Maps representing the ^13^C : ^12^C ratio were obtained by dividing the ^13^C^−^ secondary ion counts by ^12^C^−^ counts on each pixel. Similarly ^15^N : ^14^N ratios were obtained by dividing the ^12^C^15^N^−^ counts by ^12^C^14^N^−^ counts on each pixel. Numerical ^13^C : ^12^C and ^15^N : ^14^N ratio data were extracted directly from the images by selecting regions-of-interest (ROI: discrete groups of pixels that define a particular feature), and extracting the total number of counts for the given ROI. Ratios were calibrated by taking daily measurements of the resin surrounding the sections to correct for instrumental mass fractionation (IMF). The resin was also independently analysed by IRMS (δ^13^C = −30.08, SD = 0.196; δ^15^N = −3.08, SD = 2.1; *n *=* *11). Quoted uncertainties are the standard error of the mean of the measured ROIs for any given fungal/plant cell component across all fields of views (*n *=* *30) from all root samples (*n *=* *3, and one unlabelled control).

The effect of quasi-simultaneous arrivals (QSA) (Slodzian *et al*., 2001) on the ratios was tested by applying different beta corrections to individual ROIs from several images. Beta values of 1, 0.75 and 0.5 (cf. Hillion *et al*., 2008) were applied to the data directly using the OpenMIMS software, but were found to have a negligible effect on these data (data not shown). As such, the data presented here were not corrected for QSA.

### Statistics

We used R (R 2.15.0 with package vegan 2.0-4 (http://www.r-project.org/) for multivariate correspondence analysis of C concentrations (μg biomarker-C g^−1^ soil) and ^13^C excess (ng ^13^C excess g^−1^ soil) of 18 individual PLFA and 2 NLFA biomarkers (Gonzales *et al*., 2008). SigmaPlot 12.0 was used for all other statistical analyses (Systat Software GmbH, Erkrath, Germany).

## Results

### Mycorrhizal colonisation and AM biomarkers in root- and hyphae-associated compartments

After 4 wk, wheat plants were *c*. 15 cm high and 56% of root length of plants in split-boxes were colonised by AM fungi (SE = 4.75, *n *=* *10). Viable mycorrhizal hyphae were abundant in both compartments. Biomarkers for AM fungi (PLFA: 16:1ω5, NLFA: 16:1ω5 and 18:1ω7) were abundant in both root- and hyphae-associated compartments of planted samples, but were absent from unplanted controls (Table1).

### Carbon and nitrogen flow within plant cells and AM intraradical hyphae

**Figure 2:**
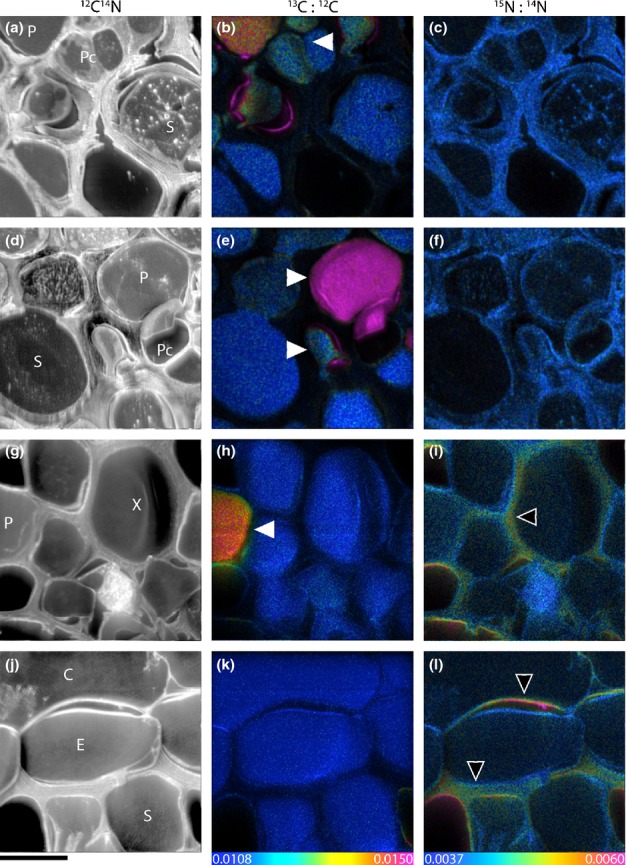
NanoSIMS images visualising transport of photoassimilated ^13^C (from ^13^CO_2_) in addition to ^15^N uptake (from ^15^NH_4_^+^) through the root stele of a mature fine root of wheat (*Triticum aestivum*). Root cell structure is visible in the greyscale ^12^C^14^N images (a, d, g, j), with corresponding ^13^C : ^12^C (b, e, h, k) and ^15^N : ^14^N (c, f, i, l) images reflecting levels of ^13^C and ^15^N enrichment, respectively. Enrichment levels are shown as hue-saturation intensity (HSI) images. The HSI colour scale for all ^13^C : ^12^C images ranges from natural abundance (^13^C : ^12^C = 0.0108, blue) to enriched (^13^C : ^12^C = 0.0150, pink). For all ^15^N : ^14^N images the scale ranges from natural abundance (^15^N : ^14^N = 0.0037, blue) to enriched (^15^N : ^14^N = 0.0060, pink). P, phloem; Pc, phloem companion cells; S, stele; E, endodermis; C, cortex; X, xylem. Areas of ^13^C enrichment are concentrated within the phloem region (white arrows), whereas ^15^N enrichment is commonly localized within the cell walls (black arrows). Bars, 10 μm (for all images).

**Figure 3:**
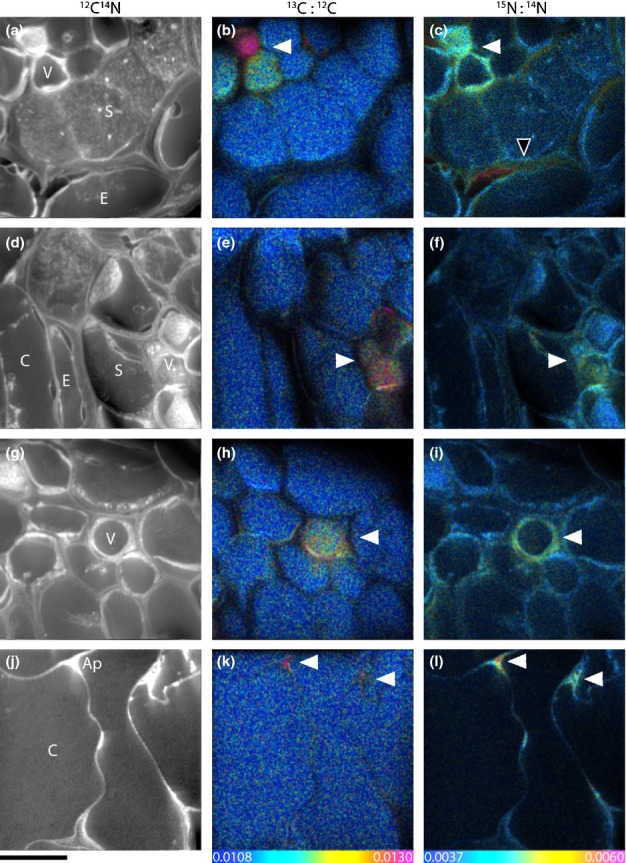
NanoSIMS images depicting photoassimilated ^13^C (from ^13^CO_2_) in addition to ^15^N uptake (from ^15^NH_4_^+^) by root cells and arbuscular mycorrhiza in a not yet fully developed fine root (i.e. close to the root tip) of wheat (*Triticum aestivum*). Root cell structure is visible in the greyscale ^12^C^14^N images (a, d, g, j), with corresponding ^13^C : ^12^C (b, e, h, k) and ^15^N : ^14^N (c, f, i, l) images reflecting levels of ^13^C and ^15^N enrichment, respectively. Enrichment levels are shown as hue-saturation-intensity (HSI) images. The HSI colour scale for all ^13^C : ^12^C images ranges from natural abundance (^13^C : ^12^C = 0.0108, blue) to enriched (^13^C : ^12^C = 0.0130, pink). For all ^15^N : ^14^N images the scale ranges from natural abundance (^15^N : ^14^N = 0.0037, blue) to enriched (^15^N : ^14^N = 0.0060, pink). V, vascular cells; S, stele; E, endodermis; C, cortex; Ap, apoplast. ^13^C enrichment spatially co-occurred with ^15^N enrichment (white arrows). Additional ^15^N enrichment is also seen in the cell walls (black arrow). Bars, 10 μm (for all images).

**Figure 4:**
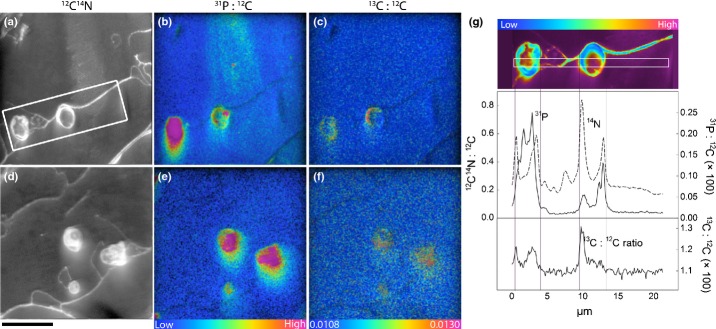
NanoSIMS images showing the uptake of photoassimilated ^13^C into intraradical arbuscular mycorrhizal hyphae within the root cortex of mature fine roots of wheat (*Triticum aestivum*) after 8 h of ^13^CO_2_ labelling. Root and mycorrhizal cell structure is visible in the greyscale ^12^C^14^N images (a, d), with corresponding ^31^P : ^12^C (b, e) and ^13^C : ^12^C (c, f) images reflecting levels of ^31^P and ^13^C enrichment, respectively. Enrichment levels are shown as hue-saturation intensity (HSI) images. The HSI colour scale for all ^31^P : ^12^C images represent low (blue) to high (pink). For all ^13^C : ^12^C images the scale ranges from natural abundance (^13^C : ^12^C = 0.0108, blue) to enriched (^13^C : ^12^C = 0.0130, pink). A line profile through the root and hyphal cells (g) (region defined in (a)), shows that within the fungal hyphae P and N concentrations are high, and ^13^C enrichment is evident. Bars, 10 μm (for all images).

**Figure 5:**
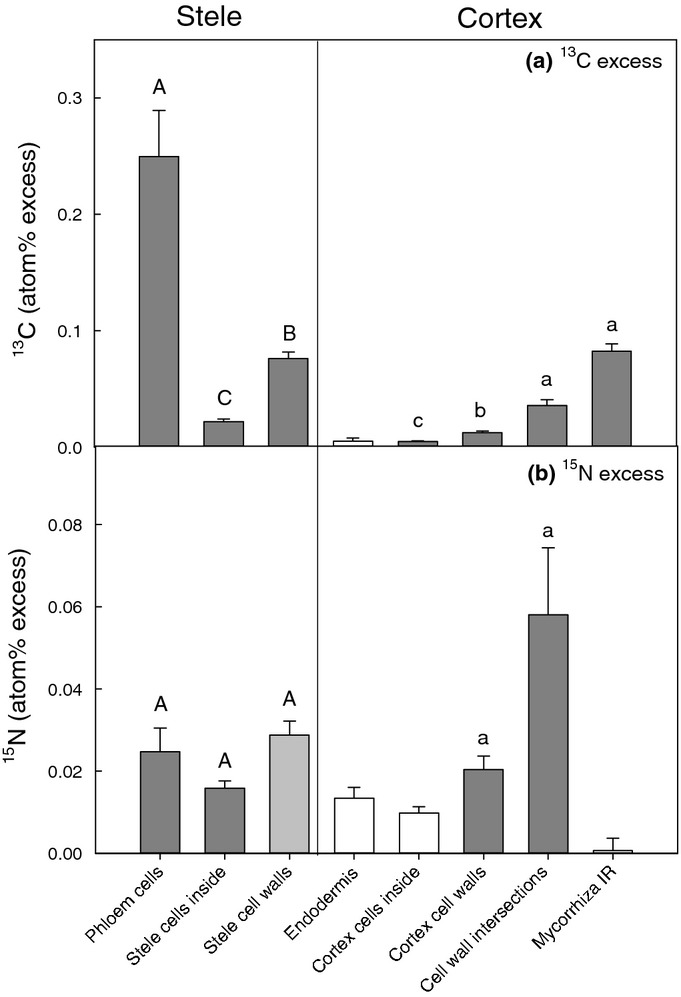
^13^C and ^15^N atom% excess in cellular and subcellular compartments of wheat (*Triticum aestivum*) fine roots, associated with arbuscular mycorrhizal fungi, after 4 h of ^13^CO_2_ labelling and 2 h after ^15^NH_4_^+^ addition to the soil. ^13^C : ^12^C and ^15^N : ^14^N ratios of specific compartments of root cross-sections were obtained from NanoSIMS images by extracting ^13^C/^12^C and ^12^C^15^N/^12^C^14^N secondary ion counts of selected regions-of-interests (ROI). Enrichment of ^13^C or ^15^N (atom% excess, APE) was calculated for each ROI by subtracting mean atom% ^13^C or ^15^N of corresponding ROIs from an unlabelled control root (describing the same cellular feature), from atom% ^13^C or ^15^N of the given ROI. Bars show the mean APE of all ROIs of a specific root compartment across different root samples (7–9 NanoSIMS images per root, three labelled root samples, total number of ROIs: 569). Mycorrhiza IR, mycorrhizal intraradical hyphae. Closed bars indicate plant compartments that are significantly isotopically enriched compared to the unlabelled root control (dark grey: *P *<* *0.001, light grey: *P *<* *0.05), whereas open bars are not significantly isotopically enriched. Differences among isotopic-enriched plant compartments have been analysed separately for Stele and Cortex with rank-based ANOVA (Kruskal–Wallis) followed by pairwise multiple comparison (Dunn's method). There was a significant influence of plant compartment on ^13^C-enrichment in both Stele and Cortex (*P *<* *0.001), but not on ^15^N enrichment. Pairwise differences between root compartments (*P *<* *0.05) are indicated by capital or small letters for Stele and Cortex, respectively. Error bars, + SE.

Isotope ratio images enabled visualisation of the *in situ* flow of recent photosynthates and N through a plant root and its associated AM structures (Figs2–4). We did not encounter mycorrhizal arbuscules in the samples we processed for NanoSIMS analysis, but we did identify mycorrhizal intraradical hyphae. After 8 h of ^13^CO_2_ labelling, phloem cells were significantly enriched in ^13^C compared to other areas of the root stele (Figs2, 3). Apart from the phloem, ^13^C enrichment also occasionally appeared in apoplastic regions of the stele (i.e. cell walls), but not within the cytoplasm of other cells (Figs2,5). In the root cortex, labelled ^13^C occurred predominantly in AM intraradical hyphae, with lower amounts also detected in cortex cell walls. This ^13^C was commonly located at intersections of more than two cells (Figs3, 4), but not in symplastic areas of the cortex. The ^15^N taken up from the soil solution was detected in both cells and cell walls of the stele (symplast and apoplast), but was only observed in cell walls in the cortex (apoplast only) (Fig.5). AM intraradical mycorrhizal hyphae did not incorporate ^15^N.

There was a qualitative difference in the pathways of photosynthates and N through root sections close to the root tip compared to more developed fine root sections. Root sections close to the root tip were characterised by immature sieve tube and xylem elements and a stele diameter < 100 μm (Fig. S2). In these root tip sections ^13^C and ^15^N were significantly co-located (Fig.3, *R*^2^ = 0.41 and 0.47, *P *<* *0.001, for stele and cortex areas). By contrast, mature root sections, which were characterised by a central metaxylem vessel, clearly visible protoxylem and phloem cells, as well as mycorrhizal intraradical hyphae in the root cortex (Fig.1), ^13^C and ^15^N were not correlated in the stele or in the cortex. Additionally, in the cortex of immature roots both ^13^C and ^15^N enrichment occurred mainly in widened (apoplastic) intersections between more than two cells (Fig.3k,l), whereas in mature roots ^13^C-enrichment was located primarily in intraradical mycorrhizal hyphae (Fig.4).

As expected, intraradical mycorrhizal hyphae were rich in phosphorus (P) (Fig.4b,e,g). Although the ^13^C signal within the intraradical hyphae cross-section was predominantly linked to the N-rich interface between plant and fungal cells (in between fungal and plant plasma membrane, Fig.4c,f,g), P was not restricted to that area and occurred also in the inner part of the intraradical mycorrhizal hyphae.

### Belowground C allocation into roots, and root- and hyphae-associated microbes

Approximately 75% of the ^13^C injected during the labelling period was recovered in the plant and microbial biomass of the harvested split-boxes (after 4 h: a total of 94 mg ^13^C excess was recovered in plant and soil pools of 35 split-boxes, with a total of 125 mg ^13^C added, Table2).

**Table 2:** Partitioning of plant-assimilated carbon (C) in aboveground-- and belowground pools of wheat (*Triticum aestivum*) associated with arbuscular mycorrhizal fungi 24 h after the start of an 8-h ^13^CO_2_ labelling period

	Shoot		Root		Root-accessible				Hyphae-accessible			
	Bulk tissue		Bulk tissue		DOC		Microbial biomass		DOC		Microbial biomass	
^13^C atom percent excess (APE)												
	APE (%)	*n*	APE (%)	*n*	APE (%)	*n*	APE (%)	*n*	APE (%)	*n*	APE (%)	*n*
4 h	1.87 (0.09)***	12	0.38 (0.02)***	13	−0.001 (0.000)	9	0.052 (0.01)**	9	−0.001 (0.000)	9	0.119 (0.05)**	8
8 h	2.32 (0.11)***	10	0.60 (0.04)***	11	0.002 (0.001)	8	0.156 (0.06)**	8	−0.002 (0.00)	8	0.015 (0.01)*	8
24 h	1.78 (0.15)***	10	0.80 (0.08)***	10	0.005 (0.002)*	7	0.114 (0.03)*	4	−0.000 (0.001)	8	0.091 (0.03)*	6

Carbon content												
	mg C split-box^−1^	*n*	mg C split-box^−1^	*n*	μg C g^−1^ soil	*n*	μg C g^−1^ soil	*n*	μg C g^−1^ soil	*n*	μg C g^−1^ soil	*n*
4 h	132.0 (8.4)	12	84.2 (10.6)	13	166.4 (14.2)	9	81.8 (13.4)	9	153.9 (8.2)	9	57.3 (12.0)	8
8 h	126.5 (7.6)	10	78.8 (8.7)	11	88.3 (13.6)	8	87.1 (19.4)	8	102.7 (9.0)	8	51.4 (8.0)	8
24 h	116.3 (7.1)	10	108.2 (13.7)	10	88.0 (8.2)	7	78.2 (42.2)	4	101.6 (4.4)	8	46.9 (21.3)	6

Amount of plant-assimilated ^13^C												
	μg excess ^13^C split-box^−1^	*n*	mg excess ^13^C split-box^−1^	*n*	μg excess ^13^C split-box^−1^	*n*	μg excess ^13^C split-box^−1^	*n*	μg excess ^13^C split-box^−1^	*n*	μg excess ^13^C split-box^−1^	*n*
4 h	2365 (148)	12	314.5 (33)	13	–	–	17.21 (5.3)	8	–	–	16.96 (8.5)	8
8 h	2944 (205)	10	474.5 (40)	11	–	–	37.20 (12.1)	8	–	–	2.62 (0.5)	7
24 h	1929 (88)	10	800.5 (60)	10	1.85 (0.7)	7	37.40 (17.7)	4	–	–	14.3 (12.1)	5

Partitioning of recently plant-assimilated C in above- and belowground pools																
	Total amount of excess ^13^C		Fraction of total amount (%)				Fraction of total belowground amount (%)									
	mg excess ^13^C split-box^−1^	*n*	Above-ground	*n*	Below-ground	*n*	Roots	*n*	Root-asc. microbes	*n*	Hyph-asc. microbes	*n*	Root-asc. DOC	*n*	Hyph-asc. DOC	*n*
4 h	2.7 (0.2)	12	87.0 (0.9)	12	13.0 (1.0)	12	91.2 (1.3)	13	6.0 (1.6)	8	4.0 (1.5)	8	0.0 (0.0)	9	0.0 (0.0)	8
8 h	3.5 (0.2)	10	85.1 (0.8)	10	14.9 (0.8)	10	89.4 (2.3)	10	9.1 (3.4)	7	0.4 (0.1)	6	0.0 (0.0)	8	0.0 (0.0)	8
24 h	2.7 (0.1)	10	70.1 (1.6)	10	29.8 (1.6)	10	96.9 (1.1)	10	4.3 (2.0)	4	1.8 (1.6)	4	0.26 (0.1)	7	0.0 (0.0)	8

DOC, dissolved organic carbon; Root-asc, root-associated; Hyph-asc, hyphae-associated. APE, Atom Percent Excess. Values are means of *n* replicate samples, standard errors are given in brackets. ^13^C enrichment of samples that were significantly different from the unlabelled control are highlighted in bold (

* ,*P *<* *0.05;

** , *P *<* *0.01;

*** , *P *<* *0.001).

This analysis also includes samples where NH_4_^+^ had been added to the soil. NH_4_^+^ addition slightly increased ^13^C uptake in the microbial biomass pools, but this was not statistically significant. Total amount of excess ^13^C has been calculated separately for each pool (μg g^−1^ soil or plant biomass) and then extrapolated for each split-box to show the total flow of C per plant. Aboveground (= shoots) and belowground (= roots, microbial biomass and DOC) pools are presented as percentages of the total amount per split-box. Roots, microbes, and DOC are additionally given as percentages of the total belowground amount of excess ^13^C.

Analysis of ^13^C in plants, soil microbial biomass and extractable DOC showed that photosynthetic C was rapidly transported belowground. After 4 h, 13% of total ^13^C assimilated by plants was found in root and soil microbial pools (Table2). From this C, 90% was stored in roots whereas 10% was found in the soil microbial biomass (6% and 4% in root- and hyphae-associated microbes, respectively). Twenty-four hours after the start of the 8-h labelling period, a total of 29% of the photoassimilated ^13^C was found in belowground pools.

Plant-assimilated C was transferred to the soil microbial community from either roots or hyphae without being released to the DOC pool in the soil solution. The microbial biomass in both compartments was significantly enriched in ^13^C after only 4 h of ^13^CO_2_ labelling. However, there was no significant excess of ^13^C in the extractable DOC pool in either compartment at 4 or 8 h after the start of ^13^CO_2_ labelling. Only a small enrichment in DOC of the root compartment was detected after 24 h (Table2).

### Different belowground dynamics in root- vs hyphae-associated communities

**Figure 6:**
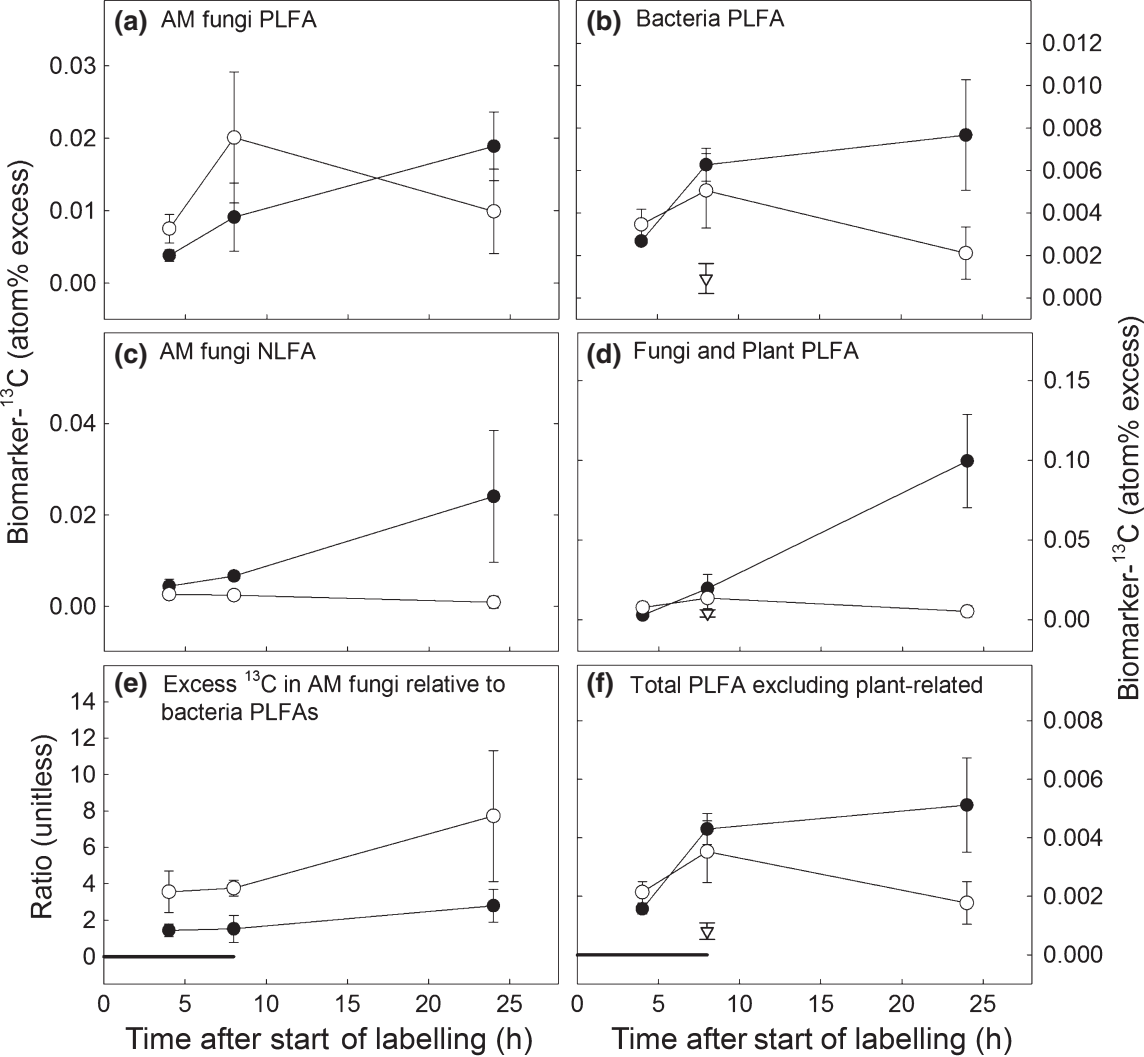
Dynamics of C recently assimilated by wheat (*Triticum aestivum*) plants through root- and arbuscular mycorrhizal hyphae-associated soil microbial communities. Presented is atom% excess of ^13^C in phospholipid fatty acid (PLFA) and neutral lipid fatty acid (NLFA) biomarkers of fungi, bacteria and AM fungi sampled 4, 8 and 24 h after the start of the 8 h ^13^CO_2_ labelling period. Each circle displays the average of replicate samples (*n *=* *4, error bars = ± 1 SE). Open circles, harvested from hyphae-associated compartment of the split-box; closed circles, from root-associated compartment of the split-box. Grey triangle, harvested from unplanted control split-boxes after 8 h of ^13^CO_2_ labelling. AM fungi, arbuscular mycorrhizal fungi. (a) PLFA biomarker used for AM fungi: 16:1ω5. (c) NLFA biomarker used for AM fungi: 16:1ω5 and 18:1ω7. For specific bacterial and fungal/plant group biomarkers (b and d, respectively) see Table1. (f) Sum of all PLFAs (as defined in Table1) excluding 18:1ω6 and 18:2ω6,9 that can potentially also occur in plant remains. Horizontal line in (e) and (f) marks the time period where split-boxes were exposed to ^13^CO_2_. For statistical analysis of the data see Table3. The figure shows a qualitative difference in turnover of plant-derived C in hyphae- and root-associated communities. Although the ^13^C signal is still increasing in root-associated communities after the end of the labelling period, it declines in hyphae-associated communities.

^13^C in PLFA biomarkers showed different temporal dynamics in root- and hyphae- compartments. The ^13^C in microbial PLFAs of hyphae compartments increased quickly after the start of photosynthetic labelling being similar to, or higher than, ^13^C in root-associated PLFAs after 4 h (Fig.6b,f). These then decreased close to initial concentrations some 16 h after the end of the labelling period (Fig.6; Table3). By contrast, ^13^C in root compartments continued to accumulate in PLFAs/NLFAs after the end of the labelling period (Fig.6; Table3). The strongest increase in ^13^C after the labelling period in root-associated soil microbial communities was found in the saprotrophic fungi biomarker 18:2ω6,9 (Fig.6d). The ratio of excess ^13^C in the AM fungal PLFA biomarker (16:1ω5) to excess ^13^C in bacterial PLFA biomarkers was higher in hyphae compartments compared to root compartments (Fig.6e). There was no difference between compartments in the ratio of total C concentrations of PLFA 16:1ω5 to bacterial biomarkers (Table1).

**Table 3:** Effects of time and compartment type (root- or arbuscular mycorrhizal hyphae-associated soil) on uptake of photoassimilated ^13^C into phospho- and neutral lipid fatty acids (PLFA and NLFA) in the soil of wheat (*Triticum aestivum*) planted split-boxes

	Two-way ANOVA			Pairwise multiple comparisons (*P *<* *0.05)				
at% ^13^C-Excess in PLFA/NLFA biomarkers of	Time	Comp.	Time × Comp.	Time (4–8–24 h)		Comp. after 4 h	Comp. after 8 h	Comp. after 24 h
				Root-associated comp.	Hyphae-associated comp.			
Bacteria (PLFA)	0.08	0.054	**0.046**	**a-ab-b**	a-a-a	0.59	0.49	**0.008**
Fungi/Plant (PLFA)	**< 0.001**	**0.001**	**< 0.001**	**a-b-c**	a-a-a	0.37	0.58	**< 0.001**
Total PLFAs excluding plant-related	**0.017**	0.060	**0.042**	**a-b-b**	a-a-a	0.52	0.47	**0.008**
AM fungi (PLFA)	0.087	0.615	0.138	a-a-a	a-a-a	–	–	**–**
AM fungi (NLFA)	0.725	**0.003**	**0.018**	a-a-a	a-a-a	0.839	0.216	**< 0.001**
Excess ^13^C in AM fungi relative to bacteria PLFAs	0.052	**0.004**	0.760	a-a-a	a-a-a	**0.02**	0.25	**0.045**

We analysed the weighted average of at% ^13^C-excess for groups of PLFA and NLFA biomarkers by a two-way ANOVA with time (4, 8 and 24 h after the start of the 8 h ^13^CO_2_ labelling period) and compartment (root- or hyphae-associated compartment of the split-box) as factors, followed by pairwise multiple comparisons (*P *<* *0.05, Holm-Sidak method). Bold numbers indicate statistically significant differences (*P *<* *0.05). ‘Total PLFAs excluding plant-related’ means excluding 18:1ω6 and 18:2ω6,9 that can potentially also occur in plant remains. PLFA biomarker used for arbuscular mycorrhizal (AM) fungi: 16:1ω5; NLFA biomarker used for AM fungi: 16:1ω5 and 18:1ω7. For other group biomarkers see Table1. For presentation of means see Fig.6.

The addition of ^15^NH_4_^+^ to either the root- or hyphae-compartment generally increased the fraction of ^13^C in PLFA and NLFA biomarkers after 24 h. ^13^C atom% excess of the AM fungal biomarker PLFA 16:1ω5 was 0.026 (± 0.010 SE) and 0.010 (± 0.006 SE) with and without N addition, respectively, in the hyphae-associated compartments. In root-associated compartments this excess was 0.039 (± 0.012 SE) and 0.019 (± 0.004 SE) with and without N addition, respectively. Although almost all PLFA and NLFAs showed increased concentrations after N addition (data not shown), most of these trends were statistically insignificant, except for NLFA AM fungal biomarkers in the hyphae-associated compartment (0.117 (± 0.033 SE) and 0.015 (± 0.024 SE) with and without N addition, respectively (*P *<* *0.05, *n *=* *4)).

### Different microbial groups utilise C in root- vs hyphae-associated communities

**Figure 7:**
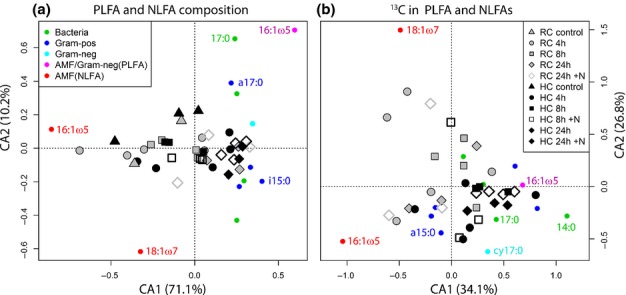
Correspondence analysis (CA) based on the absolute carbon C concentration (a, μg biomarker-C g^−1^ soil) or the concentration of ^13^C in excess (b, ng ^13^C excess g^−1^ soil) of phospho- and neutral lipid fatty acids (PLFA and NLFA) biomarkers extracted from soil associated with wheat (*Triticum aestivum*) roots or associated arbuscular mycorrhizal hyphae. PLFA biomarkers 18:1ω6 and 18:2ω6,9 that are known to also occur in plants were excluded from the analysis. RC, root-associated compartment; HC, hyphae-associated compartment of split-boxes. AMF, arbuscular mycorrhizal fungi. Individual PLFA and NLFA biomarkers are depicted as small coloured circles, indicating microbial groups based on their influence on the two ordinates. Some biomarkers are exemplarily identified by their fatty acid denotation (e.g. 16:1ω5). This analysis shows that although bulk microbial communities in root- and hyphae-associated soil are similar (a), plant-derived C is processed by distinct fractions of the microbial community in root- and hyphae-associated soil (b).

A multivariate correspondence analysis based on excess-^13^C concentrations in 13 PLFA and 2 NLFA biomarkers (excluding potentially plant-born biomarkers 18:1ω6 and 18:2ω6,9) revealed that different microbes were involved in the uptake and turnover of plant C in root- and hyphae-associated microbial communities (Fig.7). Although the microbial community composition *per se* was similar in root and hyphae compartments, the part of the community utilising recent plant C differed (Fig.7). In hyphae-associated microbial communities the uptake of recent plant ^13^C was associated to a greater extent with the PLFAs 16:1ω5 (that occur in both AM fungi and Gram-negative bacteria), general bacteria and Gram-negative bacteria biomarkers. By contrast, ^13^C uptake in root-associated communities were largely dominated by NLFA biomarkers 16:1ω5 and 18:1ω7 (specific for AM fungi), and some Gram-positive biomarkers (Fig.7). When 18:1ω6 and 18:2ω6,9 were included in the analysis it appears that ^13^C-labelled 18:2ω6,9 is clearly more abundant in root-associated than in hyphae-associated PLFAs, especially after 24 h (Fig. S3b). This, however, is not the case for the abundance of unlabelled 18:2ω6,9 (Fig. S3a). When both potentially plant-borne PLFA biomarkers and the NLFAs 16:1ω5 and 18:1ω7 (that may also occur in mycorrhizal intraradical hyphae inside roots) were excluded from the multivariate analysis, there is a less pronounced, but still notable difference between ^13^C feeding communities in root and hyphae compartments (Fig. S3c,d).

## Discussion

A rapid and close coupling between plant photosynthesis and belowground C allocation to roots and subsequent soil respiration has repeatedly been demonstrated (Johnson *et al*., 2002; Dilkes *et al*., 2004; Bahn *et al*., 2009, 2013). AM fungi are known to act as a major sink for plant photosynthates over time periods of days to weeks (Johnson *et al*., 2002; Olsson & Johnson, 2005; Drigo *et al*., 2010). The possible role of AM fungi to act as a gateway for direct delivery of recent plant photosynthates to other soil microbes on a rapid timescale (i.e. via hyphal exudates, not by hyphae turnover) has seldom been studied due to practical difficulties in separating fungal hyphae exudates and root exudates (Olsson & Johnson, 2005; Drigo *et al*., 2010). AM fungi that provide plant photosynthates to hyphosphere microbes may be rewarded with increased nutrient availability through stimulation of microbial soil organic matter depolymerisation (Hodge *et al*., 2010; Jansa *et al*., 2013). This seems a reasonable strategy for AM fungi that lack, in contrast to other types of mycorrhiza, an ability to produce extracellular enzymes for degrading complex organic compounds (Smith & Smith, 2011). In addition, mycorrhizal fungi may have a particularly high demand for nutrients, both for their own use and as an exchange for plant C. To better understand the potential role of AM fungi as a possible ‘primer’ of microbial soil organic matter decomposition (Cheng *et al*., 2012), it is important to elucidate the pathways of C release in the plant root via direct root exudations and mycorrhiza.

### Carbon and nitrogen flow through plant root cells and mycorrhizal intraradical hyphae

Our results indicate that recently photoassimilated C is released from phloem (or phloem-adjacent cells) into the apoplast without further involvement of symplastic areas in the stele. In areas of root without a blocking endodermal layer (i.e. in immature regions close to the root tip), this release consequently allows C to further diffuse to the root's surface. This finding supports an earlier proposed hypothesis concerning root exudation being a passive loss of C by plant roots (Farrar *et al*., 2003; Jones *et al*., 2009). However, before C arrives at the ‘leaky’ immature root area it needs to pass the mature root zone. Here, the Casparian strip would block any diffusional loss from the apoplast of the stele. Movement of C into the root cortex would thus require symplastic (i.e. controlled) transport across the endodermis. At the same time this zone is usually colonised by mycorrhizal intraradical hyphae (Smith & Smith, 2011). After a few hours of ^13^CO_2_ labelling, we found mycorrhizal intraradical hyphae to be clear hot-spots of ^13^C enrichment within the root cortex. Although we found significant ^13^C enrichment of both phloem cells and mycorrhizal intraradical hyphae in the same samples, we were not able to identify where exactly C was transferred from the root to the fungus. There was no evidence that the ^13^C in the intraradical hyphae had been taken up from surrounding root cells, which were not enriched in ^13^C. Recent plant C in the mycorrhizal intraradical hyphae resided predominantly in the N-rich apoplastic area of the symbiotic interface between plant and fungus, but not in the P-rich fungal compartment in the centre (Fig.4g). This could indicate that the apoplastic part of the interface plays a role for the transport of recent plant C within the mycorrhizal intraradical hyphae. Plant-assimilated ^13^C may have been transferred to the mycorrhizal interface elsewhere, that is in mycorrhizal arbuscules (which we did not encounter in our samples) or in other places of the intraradical hyphae network. Our results indicate that a significant part of recent photoassimilates may be diverted into mycorrhizal intraradical hyphae even before they reach the passive zone of root exudation at the root tip. It is interesting to note that C transfer to the fungi is most likely under plant control as it requires symplastic transport across the endodermis and transfer to mycorrhizal intraradical structures (whereas root exudation may not be).

We did not encounter mycorrhizal arbuscules in our root samples and this is likely due to the fact that we were only able to sample and analyse a tiny fraction of the overall root system. Similarly, we may not have detected ^15^N in mycorrhizal intraradical hyphae because the dropwise addition of ^15^N to only one of the two compartments may have led to heterogeneous distribution of ^15^N within the root-mycorrhiza system. Despite these limitations, our results provide the first glimpse into C and N flow across root and mycorrhiza. We present visualisation of the *in situ* flow of labile C compounds derived from photosynthesis of ^13^CO_2_ through plant roots and arbuscular mycorrhizal features. Further studies are now required to explore the potential of NanoSIMS to investigate the exchange of C and N between plant and mycorrhizal fungi, ideally also including mycorrhizal arbuscules.

### Rapid transfer of recent photoassimilates to root- and hyphosphere soil microbes

Highly enriched intraradical mycorrhizal features in mature root sections indicate that the mycorrhizal hyphae network may be an important gateway for photosynthetic C in the short term. This was further supported by the finding that both ^13^C-PLFA and ^13^C-microbial biomass fumigation extraction analyses showed similar total amounts of ^13^C in the microbial biomass of root- and hyphae-compartments in the short term. For example, after 8 h totals of 1.32 ± 0.49 and 1.76 ± 0.36 ng excess ^13^C g^−1^ dry soil were stored in PLFAs of hyphae- and root-associated soil, respectively (Fig. S4, Table S1). Both PLFAs and bacteria-specific biomarkers also showed similar total amounts of ^13^C in both compartments 4 and 8 h after the start of labelling (Figs6, S4; Table S1). This indicates a rapid transfer of ^13^C not only from the root, but also from hyphae to soil microbes. Although some studies have shown that AM hyphae exude plant-derived C into the hyphosphere (Johansson *et al*., 2004; Toljander *et al*., 2007; Cheng *et al*., 2012), there is no evidence for the direct transfer of recent photoassimilates from mycorrhizal hyphae to hypersymbiont microbes in the surrounding hyphosphere (Jansa *et al*., 2013). Although we cannot rule out that C transfer to soil microbes could have happened via hyphae turnover, our results strongly indicate that transfer was via hyphal exudation, due to the short incubation time.

The lack of ^13^C in the DOC of the soil solution of both plant root- and hyphal-compartments (Table2) indicates rapid uptake of root- or hyphae-exuded C by surrounding soil microbes. The surface of both plant roots (Watt *et al*., 2006) and mycorrhizal extraradical hyphae (Scheublin *et al*., 2010) are colonised by specific assemblages of bacterial species that differ from the bulk soil bacterial community. This may explain why, overall, microbial communities were similar in root- and hyphae-associated soil, but the fraction of the community utilising root- or hyphae-released C was different (Fig.7). It is therefore reasonable to assume that different species assemblages are associated with fine plant roots and AM hyphae.

In contrast to PLFA biomarkers, NLFA biomarkers for AM fungi showed consistently higher ^13^C enrichment in root compartments than in hyphae compartments after 4 and 8 h of labelling (Fig.6). A possible explanation is that the NLFA biomarker 16:1ω5 occurs in high numbers in mycorrhizal intraradical features and in spores (Olsson & Johnson, 2005). A short-term increase of ^13^C in NLFAs of root-associated soil samples may reflect contamination of the soil with fractions of fine roots containing mycorrhizal intraradical hyphae, whereas the incorporation of ^13^C in spores may only happen at longer timescales.

### Is the hyphal pathway more dynamic?

Unlike in root compartments, ^13^C in bacterial PLFAs of hyphae-compartments almost returned to initial concentrations 16 h after the end of the labelling period. This indicates that plant-derived C exhibits a faster turnover in microbial PLFA biomarkers of hyphae-associated soil than in root-associated soil. Mycorrhizal hyphae seem to preferably transport recently assimilated (i.e. only a few hours old) labile plant C, which were rapidly replaced by nonlabelled components after the labelling period. Plant roots, by contrast, seem to have continued to deliver C that was photosynthesised several hours before, most likely in the form of more complex compounds such as mucilage or root border cap cells, which are naturally not transported through mycorrhizal hyphae. A significant fraction of the C photoassimilated by plants during daytime is stored as starch, from which it is mobilised during the subsequent night as a substrate for aboveground and belowground growth and respiration (Bahn *et al*., 2009; Graf & Smith, 2011). The finding that less of the ^13^C signal was found in microbial communities of hyphae compartments compared to root compartments after 24 h (i.e. after the subsequent night after ^13^CO_2_-labelling) may thus also indicate that the hyphal pathway is less important for night-time belowground C transport. More research is needed to explore the possible different dynamics of C being transported via the root and the hyphal pathway to soil microbes.

The particularly strong increase of ^13^C in the PLFA 18:2ω6,9 in root-associated soil may indicate the feeding of saprotrophic fungi on this different C source. However, as this PLFA is also known to occur in plants this could be due to an increased contribution of ^13^C-enriched plant remains to soil samples.

### Possible role of mycorrhiza for regulating carbon transfer

NH_4_^+^ added to either the root or the hyphae compartment increased the fraction of ^13^C in AM fungi biomarkers after 24 h. Although the overall amount of NH_4_^+^ added to the soil was small (*c*. 3% of the total NH_4_^+^ in the soil solution), it may have caused localised increased NH_4_^+^ concentrations around plant roots and mycorrhizal hyphae. We speculate that his could have acted as a first signal for the encounter of an N-rich organic patch in the soil. This may have then triggered an increase of belowground plant C allocation towards that spot in order to further stimulate microbial decomposition of the encountered substrate. If root exudation was mainly a passive diffusion process driven by a lack of a Casparian strip in immature root areas as proposed (Farrar *et al*., 2003; Badri & Vivanco, 2009), well-directed priming effects induced by plants at substrate-rich spots in the soil would be impossible. Our finding that NH_4_^+^ addition to the hyphal compartment increased the amount of plant C delivered to this compartment, indicates that mycorrhizal fungi may play a key role in a controlled response of plants to varying soil nutrient content. As these results were only weakly significant, however, further studies are needed to clarify the role of mycorrhizal fungal as a potential partner for well-directed soil priming.

### Conclusion

Our findings indicate that a significant fraction of recently photoassimilated C delivered to soil microbes leaves plant roots via mycorrhizal hyphae already upstream of the place of passive root exudations. AM extraradical hyphae, which enlarge root areas, may thus not only improve nutrient uptake by plants, but also act as an effective distributor of recently assimilated plant C to the soil microbial community. Moreover, the amount of plant C channelled through the AM pathway seems to respond quickly to changing soil nutrient availability. Based on our results we propose qualitative differences between the two pathways of recent plant C release to soil microbes: hyphosphere C release is more intimately coupled to plant photosynthesis than rhizosphere C release, and processed by a different set of microbes. Furthermore, the involvement of the mycorrhizal partner may allow for a finer regulation of plant responses to soil nutrient conditions as C is not necessarily lost passively, as likely happens through root exudation.

## Supporting Information

**Fig. S1**Experimental set up. **Fig. S2**Light micrograph of a transverse section through an immature wheat root (*Triticum aestivum)*. **Fig. S3**Correspondence analysis (CA) based on C concentration and concentration of excess ^13^C of phospho- and neutral lipid fatty acid biomarkers extracted from soil associated with wheat (*Triticum aestivum*) roots or associated arbuscular mycorrhizal hyphae. **Fig. S4**Flow of recently photoassimilated C from wheat plants (*Triticum aestivum*) into root- and arbuscular mycorrhizal hyphae-associated soil microbial communities. **Table S1**Effects of time and compartment type (root- or hyphae-associated soil) on total amounts of photoassimilated ^13^C into phospho- and neutral lipid fatty acids (PLFA and NLFA) in the soil of wheat (*Triticum aestivum*) planted split-boxes **Note S1** Justification of sample preparation method used for NanoSIMS analysis of wheat (*Triticum aestivum*) roots associated with arbuscular mycorrhizal fungi.
