# Supplementary material for: Exploring the transfer of recent plant photosynthates to soil microbes: mycorrhizal pathway vs direct root exudation
Source: New Phytol. 2014 Nov 10;205(4):1537–51. doi: 10.1111/nph.13138 (PMC4357392; doi:10.1111/nph.13138)
Supplement: Supplementary file 1 — Fig. S1Experimental set up. Fig. S2Light micrograph of a transverse section through an immature wheat root (Triticum aestivum). Fig. S3Correspondence analysis (CA) based on C concentration and concentration of excess 13C of phospho- and neutral lipid fatty acid biomarkers extracted from soil associated with wheat (Triticum aestivum) roots or associated arbuscular mycorrhizal hyphae. Fig. S4Flow of recently photoassimilated C from wheat plants (Triticum aestivum) into root- and arbuscular mycorrhizal hyphae-associated soil microbial communities. Table S1Effects of time and compartment type (root- or hyphae-associated soil) on total amounts of photoassimilated 13C into phospho- and neutral lipid fatty acids (PLFA and NLFA) in the soil of wheat (Triticum aestivum) planted split-boxes Note S1 Justification of sample preparation method used for NanoSIMS analysis of wheat (Triticum aestivum) roots associated with arbuscular mycorrhizal fungi. [file nph0205-1537-sd1.pdf]

***New Phytologist* Supporting Information**

Exploring the transfer of recent plant photosynthates to soil microbes: mycorrhizal pathway versus direct root exudation

Christina Kaiser, Matt R. Kilburn, Peta L. Clode, Lucia Fuchslueger, Marianne Koranda, John B. Cliff, Zakaria M. Solaiman and Daniel V. Murphy

Article acceptance date: 23 September 2014

The following Supporting Information is available for this article:

Fig. S1 Experimental set up.

Fig. S2 Light micrograph of a transverse section through an immature wheat root (*Triticum aestivum)*.

Fig. S3 Correspondence analysis (CA) based on C concentration and concentration of excess ^{13}C of phospho- and neutral lipid fatty acid biomarkers extracted from soil associated with wheat (*Triticum aestivum*) roots or associated arbuscular mycorrhizal hyphae.

Fig. S4 Flow of recently photoassimilated C from wheat plants (*Triticum aestivum*) into root- and arbuscular mycorrhizal hyphae-associated soil microbial communities.

Table S1: Effects of time and compartment type (root- or hyphae associated soil) on total amounts of photoassimilated ^{13}C into phospho- and neutral lipid fatty acids (PLFA and NLFA) in the soil of wheat (*Triticum aestivum*) planted split-boxes.

Notes S1 Justification of sample preparation method used for NanoSIMS analysis of wheat (*Triticum aestivum*) roots associated with arbuscular mycorrhizal fungi.

Fig. S1 Experimental set up. (a) Each split-box consisted of two compartments (dimension: 8.5 x 7 x 10 cm) that were separated by an assemblage of two membranes (mesh size: 30 µm) encapsulating a solid 1.5 mm-thick, wide-meshed plastic grid in between. The grid established a small void between compartments to prevent solution flow. Mycorrhizal hyphae, but not wheat roots, were able to grow through these membranes. (b) Each compartment was filled with 500 g of fresh soil. The soil was a coarse-textured agricultural soil routinely used for wheat production in rotation with legumes (88% quartz sand). Prior to use, the soil had been inoculated with 25 g of clipped fine root biomass to foster the establishment of plant arbuscular mycorrhizal colonisation. (c) Germinating wheat seeds (*Triticum aestivum* L. var. 'Wyalkatchem') were planted in one compartment of each box ('root compartment' i.e. contains both plant roots and mycorrhizal hyphae). The other compartment only hosted mycorrhizal hyphae without roots and was termed 'hyphae compartment'.

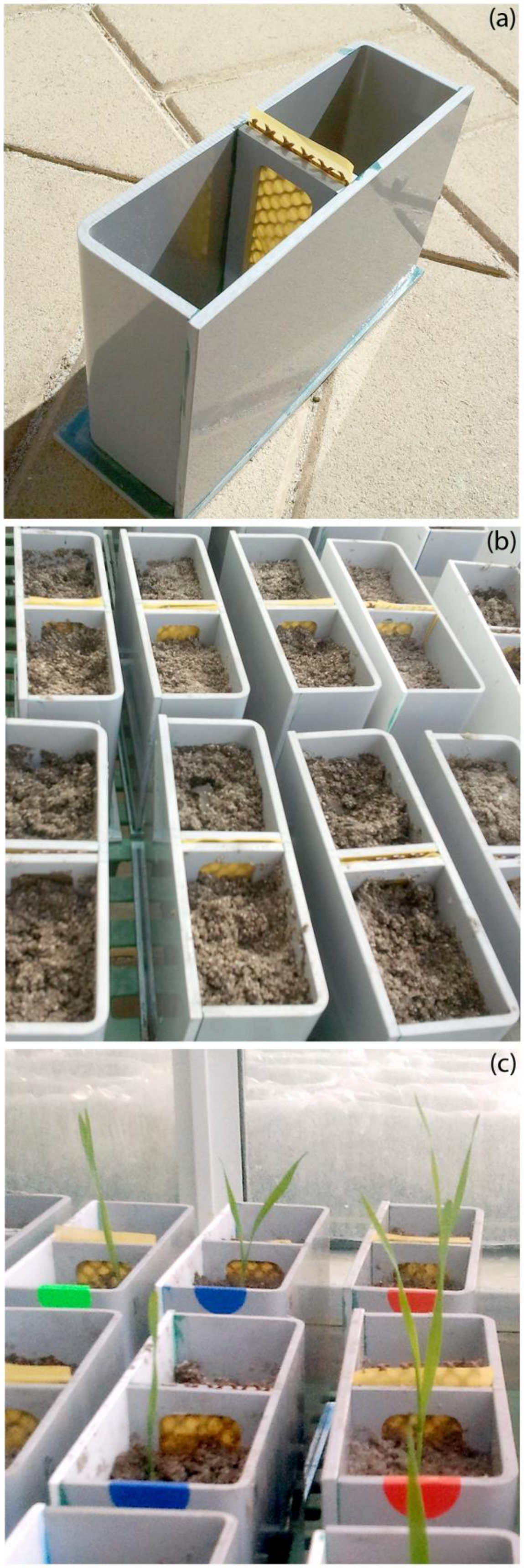
(a)
(b)
(c)

Fig. S2 Light micrograph of a transverse section through a resin-embedded fine immature wheat root (*Triticum aestivum)* , cut dry at 1 mm thickness and stained with toluidine blue. E, endodermis; S, stele; C, cortex cell layers. The square region highlighted is an example of a typical region analysed by NanoSIMS, and in this case is the same region as shown in Fig. 3(a–c). Bar, 20 µm.

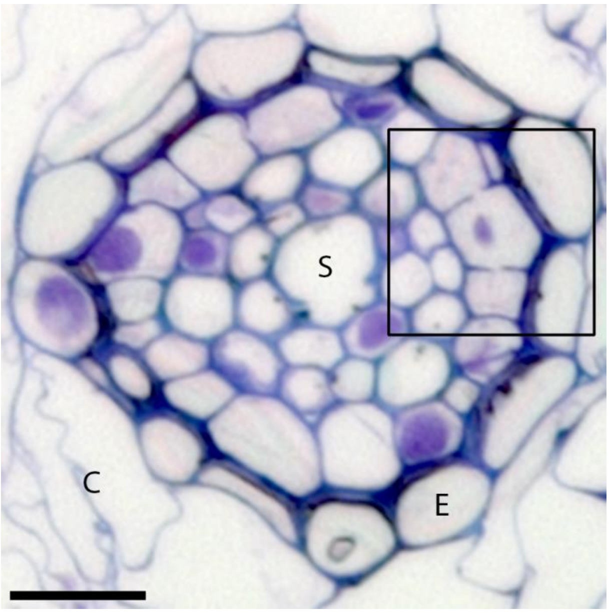

Fig. S3 Correspondence analysis (CA) based on C concentration (a and c, μg biomarker-C g^{-1} soil) and concentration of excess ^{13}C (b and d, ng ^{13}C excess g^{-1} soil) of phospho- and neutral lipid fatty acid biomarkers extracted from soil associated with wheat (*Triticum aestivum*) roots or associated arbuscular mycorrhizal hyphae. (a, b) All PLFA and NLFA biomarker as summarised in Table 1 were included in the analysis. (c, d) Biomarkers that could potentially originate from root remains in soil samples (i.e. PLFAs 18:1ω6 and 18:2ω6,9 that can also occur in plants and NLFAs 16:1ω5 and 18:2ω7 that are known to occur in mycorrhizal intraradical hyphae inside roots) were excluded. RC, root-side compartment; HC, hyphae-side compartment. Individual PLFA and NLFA biomarkers are depicted as small colored circles, indicating microbial groups based on their influence on the two ordinates. Some biomarkers are exemplarily identified by their fatty acid denotation (e.g. 16:1ω5).

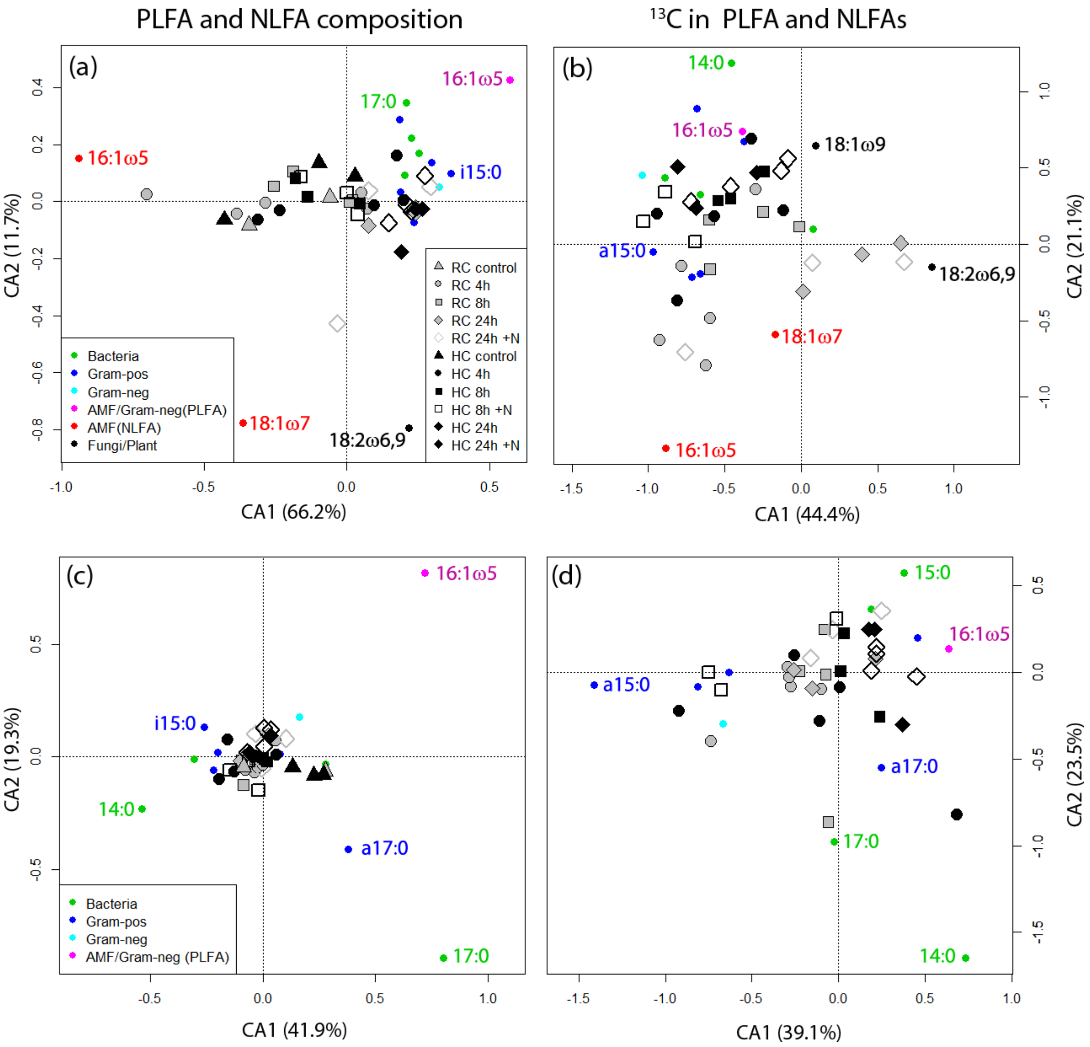
PLFA and NLFA composition
13C in PLFA and NLFAs
(a)
16:1ω5
17:0
i15:0
16:1ω5
18:1ω7
18:2ω6,9
Bacteria
Gram-pos
Gram-neg
AMF/Gram-neg(PLFA)
AMF(NLFA)
Fungi/Plant
RC control
RC 4h
RC 8h
RC 24h
RC 24h +N
HC control
HC 4h
HC 8h
HC 8h +N
HC 24h
HC 24h +N
CA1 (66.2%)
CA2 (11.7%)
(b)
14:0
16:1ω5
18:1ω9
a15:0
18:2ω6,9
18:1ω7
16:1ω5
CA1 (44.4%)
CA2 (21.1%)
(c)
16:1ω5
i15:0
14:0
a17:0
17:0
Bacteria
Gram-pos
Gram-neg
AMF/Gram-neg (PLFA)
CA1 (41.9%)
CA2 (19.3%)
(d)
15:0
16:1ω5
a15:0
a17:0
17:0
14:0
CA1 (39.1%)
CA2 (23.5%)

Fig. S4 Flow of recently photoassimilated C from wheat plants (*Triticum aestivum*) into root- and arbuscular mycorrhizal hyphae-associated soil microbial communities. Presented is total amount of excess ^{13}C (ng excess ^{13}C g^{-1} dry soil) in PLFA and NLFA biomarkers of each microbial group 4, 8 and 24 h after the start of the 8 h $^{13}CO_2$ labelling period. Each circle displays the average of four replicate samples (*n* = 4, error bars = ±1SE). Open circles: harvested from hyphae-associated compartment of the split-box, Closed circles: from root-associated compartment of the split-box. AM fungi, arbuscular mycorrhizal fungi. (a) PLFA biomarker used for AM fungi: 16:1ω5; (c) NLFA biomarker used for AM fungi: 16:1ω5 and 18:1ω7. For specific bacterial and fungal/plant group biomarkers (b and d, respectively) see Table 1. (f) Sum of all PLFAs (as defined in table 1) excluding 18:1ω6 and 18:2ω6,9 that can potentially also occur in plant remains. Horizontal line in (e) and (f) marks the time period where split-boxes were exposed to $^{13}CO_2$. For statistical analysis see Supporting information Table S1.

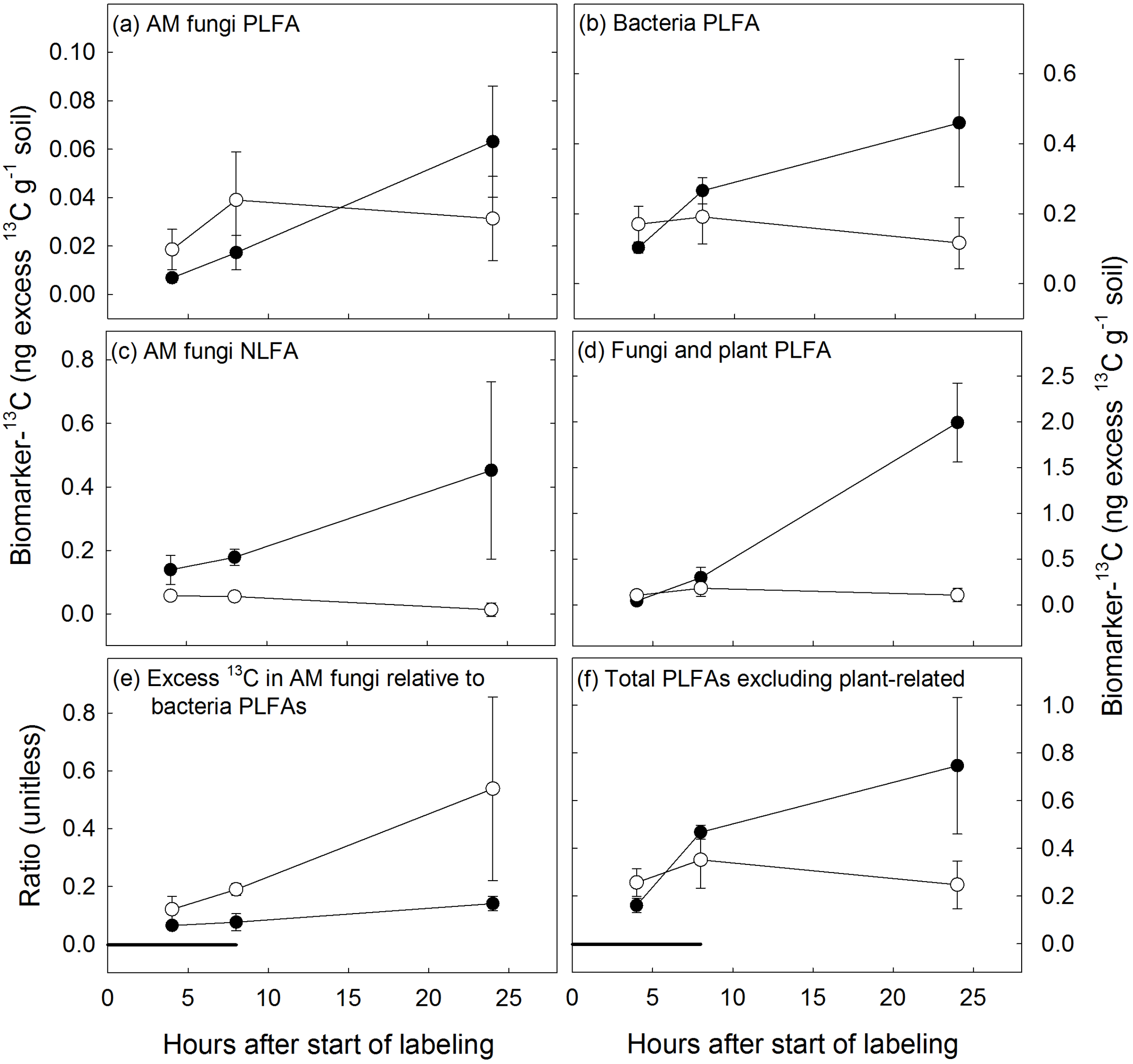

(a) AM fungi PLFA
(b) Bacteria PLFA
(c) AM fungi NLFA
(d) Fungi and plant PLFA
(e) Excess ^{13}C in AM fungi relative to bacteria PLFAs
(f) Total PLFAs excluding plant-related
Biomarker-^{13}C (ng excess ^{13}C g^{-1} soil)
Ratio (unitless)
Hours after start of labeling

Table S1 Effects of time and compartment type (root- or hyphae associated soil) on total amounts of photoassimilated ^{13}C into phospho- and neutral lipid fatty acids (PLFA and NLFA) in the soil of wheat (*Triticum aestivum*) planted split-boxes

	2-way ANOVA			Pairwise multiple comparisons (*P*<0.05)				
				Time (4-8-24 hours) within				
ng ^{13}C-Excess g^{-1} soil in PLFA/NLFA biomarkers of	Time	Comp.	Time X Comp.	Root-associated comp.	Hyphae associated comp.	Comp. after 4 h	Comp. after 8 h	Comp. after 24 h
Bacteria (PLFA)	0.133	0.071	**0.040**	**a-ab-b**	a-a-a	0.47	0.50	**0.009**
Fungi/Plant (PLFA)	**<0.001**	**0.001**	**<0.001**	**a-b-c**	a-a-a	0.34	0.40	**<0.001**
Total PLFAs excluding plant-related	**0.035**	0.068	**0.042**	**a-ab-b**	a-a-a	0.48	0.47	**0.009**
AM fungi (PLFA)	**0.024**	0.945	0.131	**a-a-b**	a-a-a	0.60	0.24	0.11
AM fungi (NLFA)	0.688	**<0.001**	**0.031**	a-a-a	a-a-a	0.233	0.082	**<0.001**
Excess ^{13}C in AM fungi relative to bacteria PLFAs	**0.042**	**0.018**	0.624	a-a-a	a-a-a	0.397	0.065	0.119

We analysed the total amount of excess ^{13}C (in ng ^{13}C excess g^{-1} soil) in groups of PLFA and NLFA biomarkers by a 2-way ANOVA with time (4, 8 and 24 h after the start of the 8 h $^{13}CO_2$ labeling period) and compartment (root- or hyphae-associated compartment of the split-box) as factors, followed by pairwise multiple comparisons (*P*<0.05, Holm-Sidak method). Bold numbers indicate statistically significant differences (*P*<0.05). 'Total PLFAs excluding plant-related' means excluding 18:1ω6 and 18:2ω6,9, which can potentially also occur in plant remains. PLFA biomarker used for AM fungi: 16:1ω5; NLFA biomarker used for AM fungi: 16:1ω5 and 18:1ω7. For other group biomarkers see Table 1. For presentation of means see Supporting Information Fig. S2.

Notes S1 Justification of sample preparation method used for NanoSIMS analysis of wheat (*Triticum aestivum*) roots associated with arbuscular mycorrhizal fungi.

The low temperature, freeze substitution method was preferred as carbon (C)-based molecules can be very difficult to immobilise and preserve. For example, only ~35% of photosynthetically fixed C preserved as protein when chemical fixation is used (Clode *et al.*, 2007). Through cross linking, this anhydrous substitution mixture stabilises and preserves cellular proteins, nucleic and fatty acids, some amino acids, and water-soluble compounds, while providing excellent structural preservation, which is needed for high resolution NanoSIMS analyses. Complex carbohydrates and lipids may not have been fully preserved, but some cross-linking and retention of these compounds still occurs. While freeze-drying represents an alternative method for preserving C- and N-based compounds in cells (Peteranderl & Lechene, 2004), highly-vacuolated root cells and water-filled vascular tissues of whole roots can suffer significantly from artefacts during freeze drying (e.g. cell shrinkage/collapse and movement/aggregation of components) (Echlin, 1992). Further, intact roots and, in general, many plant materials, are very difficult to adequately infiltrate with an epoxy resin without the use of a solvent. This necessary use of solvents during resin infiltration subsequently negates many of the advantages gained through initial freeze drying of the sample.

References

Clode PL, Stern RA, Marshall AT. **2007**. Subcellular imaging of isotopically labeled carbon compounds in a biological sample by ion microprobe (NanoSIMS). *Microscopy Research and Technique* **229**: 220–229.

Echlin P. **1992**. *Low-temperature microscopy and analysis*. New York, USA: Plenum Press.

Peteranderl R, Lechene C. **2004**. Measure of carbon and nitrogen stable isotope ratios in cultured cells. *Journal of the American Society for Mass Spectrometry* **15**: 478–485.
